# Photoluminescent Cationic Carbon Dots as efficient Non-Viral Delivery of Plasmid SOX9 and Chondrogenesis of Fibroblasts

**DOI:** 10.1038/s41598-018-25330-x

**Published:** 2018-05-04

**Authors:** Xia Cao, Jianping Wang, Wenwen Deng, Jingjing Chen, Yan Wang, Jie Zhou, Pan Du, Wenqian Xu, Qiang Wang, Qilong Wang, Qingtong Yu, Myron Spector, Jiangnan Yu, Ximing Xu

**Affiliations:** 1 Department of Pharmaceutics, School of Pharmacy, and Center for Drug/Gene Delivery and Tissue Engineering, Jiangsu University, Zhenjiang, 212001 P.R. China; 2 Department of Orthopedic Surgery, Harvard Medical School, Brigham and Women’s Hospital, 75 Francis St, Boston, MA 02115 USA

## Abstract

With the increasing demand for higher gene carrier performance, a multifunctional vector could immensely simplify gene delivery for disease treatment; nevertheless, the current non- viral vectors lack self-tracking ability. Here, a type of novel, dual-functional cationic carbon dots (CDs), produced through one-step, microwave-assisted pyrolysis of arginine and glucose, have been utilized as both a self-imaging agent and a non-viral gene vector for chondrogenesis from fibroblasts. The cationic CDs could condense the model gene plasmid SOX9 (pSOX9) to form ultra-small (10–30 nm) nanoparticles which possessed several favorable properties, including high solubility, tunable fluorescence, high yield, low cytotoxicity and outstanding biocompatibility. The MTT assay indicated that CDs/pSOX9 nanoparticles had little cytotoxicity against mouse embryonic fibroblasts (MEFs) compared to Lipofectamine2000 and PEI (25 kDa). Importantly, the CDs/pSOX9 nanoparticles with tunable fluorescence not only enabled the intracellular tracking of the nanoparticles, but also could successfully deliver the pSOX9 into MEFs with significantly high efficiency. Furthermore, the CDs/pSOX9 nanoparticles-mediated transfection of MEFs showed obvious chondrogenic differentiation. Altogether, these findings demonstrated that the CDs prepared in this study could serve as a paradigmatic example of the dual-functional reagent for both self-imaging and effective non-viral gene delivery.

## Introduction

Gene therapy has attracted considerable attention in the medical, pharmaceutical and biotechnological fields due to its potential to treat a plethora of chronic and genetic diseases^1,2^. The basic principle of gene therapy is to correct the origin of diseases *via* the delivery and subsequent expression of exogenous DNA encoding for the missing or defective gene product. In this regard, developing effective gene vectors has become essential for the improvement of gene delivery efficiency. To date, gene delivery vehicles can be classified as viral and non-viral vectors^3–5^. Generally, viral agents possess high transfection efficiency but suffer from significant limitations due to the high likelihood of mutagenesis or carcinogenesis^6^. However, these shortcomings are virtually absent in non-viral vectors such as cationic liposomes^7,8^, cationic polymers^9–11^, and inorganic nanoparticles (such as calcium phosphate nanoparticles)^12^, which offer a more efficient and safer alternative in conjunction with enhanced transfection efficiency. With the increasing demand for higher gene carrier performance, a multifunctional vector that integrates low toxicity, high transfection efficiency and bioimaging could immensely simplify gene delivery for disease treatment. Therefore, the additional benefit of bioimaging allows the self-tracking of the DNA-loaded complexes, which is desirable for gene delivery. However, the before mentioned non-viral vectors lack this self-tracking ability.

To bridge this gap, this study focuses on the utilization of fluorescent semiconductor quantum dots (QDs) for efficient non-viral gene delivery. QDs are a type of semiconductor crystal ranging from 2 to 10 nm in diameter and are one of the first nanotechnologies used in biological sciences^13–15^. Nevertheless, the most conventional semiconductor QDs contain metallic elements, which restricts their application due to concerns regarding their toxicity, stability and environmental implications^16^. Consequently, the development of benign nano-materials (*i.e*., the substitution of metals with carbon) exhibiting optical properties that are similar to those of QDs has inspired several comprehensive studies^17,18^. Admittedly, several studies have investigated CDs; however, none of these studies has reported on the direct use of these particles for gene applications^19^, with the exception of PEI modification for gene delivery^20^. Currently, the two major routes for the synthesis of CDs are top-down and bottom- up approaches. The top-down approach involves the formation of CDs from larger carbon structures *via* a post-treatment carbon breakdown process such as electrochemical oxidation^21,22^, laser ablation^23,24^ or arc discharge^25^. In the bottom-up approach, CDs are obtained from suitable molecular precursors *via* ultrasonic treatment^26^, acid dehydration^27^, thermal carbonization^28^ or combustion^6,29^. However, the major obstacles to the application of QDs are the highly demanding preparation procedures and the complex modification of these substances required for effective gene binding. To this end, this study reports the one-step synthesis of cationic CDs as nano-gene vectors from simple precursors, glucose and arginine, using microwave-assisted pyrolysis. This method is a special type of bottom-up technique that does not consume large amounts of energy and generates appreciable yields. The developed CDs were subsequently utilized to deliver a plasmid containing the gene for SOX9 into mouse embryo fibroblasts (MEFs) to investigate the potential of differentiation into chondrocytes.

The transcription factor SOX9 is a member of the SOX (SRY-type HMGbox) protein family and plays an essential role in chondrogenesis^30^ and regulates the formation of numerous cell types, tissues and organs, including hair follicles, testes and the heart^31^. SOX9 is expressed in all chondroprogenitor cells of the mouse embryo; however, its expression is abolished in hypertrophic chondrocytes and osteoblasts^32^. Previous studies have reported that SOX9 gene transfer promoted *in vitro* chondrocyte differentiation and cartilage formation; however, the vehicles used for SOX9 gene delivery were viruses, which carries potential safety risks when applied in clinical trials^33,34^. Therefore, the development of a safer and more effective vehicle to facilitate gene delivery is urgently needed. Primary MEFs, which are typically isolated from 12- to 14-day mid-gestation mouse embryos, have been previously investigated for cell commitment and differentiation into mesenchymal lineages, such as cartilage and adipose tissues^35^. Although MEFs have previously been reported to undergo chondrogenic differentiation, most of the strategies were based on viral transfection^36^ or protein factors^37,38^, which suffered from either safety issues or a lack of efficiency.

In this issue, CDs were used for the first time as a non-viral vector to deliver pSOX9 (Fig. 6) into MEFs for chondrogenic differentiation with high efficiency and low cytotoxicity, with an additional self-tracking feature for bioimaging.

## Materials and Methods

### Materials

Dulbecco’s Modified Eagle Medium/F-12 (DMEM/F12) and trypsin were purchased from Invitrogen (Invitrogen, USA). Fetal bovine serum (FBS) was purchased from Gibco (Gibco, USA). SOX9 was purchased from Bioworld Technology, Inc. (Nanjing, China). The ELISA kit for SOX9 quantification was purchased from Nanjing Qiteng Biological Co., Ltd. (Nanjing, China). MTT was purchased from Sigma (Sigma, USA). Quinine, sulfuric acid solution (H_2_SO_4_), ammonium chloride (NH_4_Cl), acrylamide, sodium orthovanadate (SOV), glucose, and colchicine were all obtained from Sinopharm Chemical Reagent Co., Ltd. (Shanghai, China). Cytochalasin D was obtained from J&K Scientific (Beijing, China).

YOYO-1 was purchased from Invitrogen (Invitrogen, USA). Arginine (ultrapure, molecular biology grade) was purchased from Sinopharm Chemical Reagent Co., Ltd. (Shanghai, China). All other reagents were purchased from Sinopharm Chemical Reagent Co., Ltd. (Shanghai, China). Principles of laboratory animal care were followed and all procedures were conducted according to the guidelines established by the National Institutes of Health, and every effort was made to minimize suffering. This study was approved by the Animal Experiment Committee of Jiangsu University.

### Cell lines

Primary MEFs were extracted from 12- to 14-day mid-gestation Kunming mice (Laboratory Animal Center of Jiangsu University, Zhenjiang, China) according to the procedures previously described^39^. The MEFs were cultured in DMEM/F12 (Gibco, USA) supplemented with 10% FBS at 37 °C in a 5% CO_2_ atmosphere.

### Preparation of carbon quantum dots

The CDs were synthesized using a microwave- assisted pyrolysis method. Briefly, arginine and glucose (0.1 g) were mixed in various weight ratios (3:1, 6:1, 9:1, 12:1, and 15:1) with 30 mL of double distilled water under ultrasonication to form a homogeneous solution which was heated with a commercial microwave oven (700 W, Galanz, China) for 10 min. After cooling to room temperature, the sample was diluted with distilled water at an appropriate ratio and dialyzed against double distilled water for 4 days. The product was concentrated, filtered through a 0.22-µm filter and freeze dried (Christ Alpha 2–4, Germany) to yield highly fluorescent CDs.

### Determination of fluorescence quantum yields

Fluorescence quantum yield is defined as the ratio of the number of radiated photons to the number of absorbed photons. In this study, the fluorescence quantum yields of CDs were determined using a comparative method. Quinine sulfate was selected as the standard due to its chemical stability, high quantum yield and lack of overlap between its excitation and emission spectra. The PL emission spectra of all of the samples were measured using an RF-5310 spectrofluorophotometer (SHIMADZU, Japan) at an excitation wavelength corresponding to the UV range. The integrated fluorescence intensity is the area under the PL curve over the wavelength range of 380 to700 nm. Absolute values were calculated according to the following equation^19^:

$${\rm{Yu}}={\rm{Ys}}\times ({\rm{Fu}}/{\rm{Fs}})\times ({\rm{As}}/{\rm{Au}}),$$

where Yu is the quantum yield of the test sample and Ys is the quantum yield of the standard sample; Fu and Fs are the integrated fluorescence intensities of the test and standard samples, respectively; and As and Au are the absorbance values of the test and standard samples, respectively.

### Preparation and characterization of CDs/pSOX9 nanoparticles

A 2 mg/mL solution of CDs was prepared using double distilled water and sterilized through a 0.22-µm filter prior to use. The CDs/pSOX9 nanoparticles were formulated at various weight ratios of CDs/pSOX9 by adding predetermined concentrations of sterilized CDs to a well-defined pDNA solution. The product was vortexed to form a homogeneous mixture. The mixtures were then incubated at room temperature for 20 min to allow for the formation of nanoparticles.

For morphological characterization, the transmission electron microscopy (TEM) images of CDs and CDs/pSOX9 nanoparticles were obtained using a JEM-2100 transmission electron microscope (JEOL, Japan) operated at an accelerating voltage of 40–100 kV. Fourier transform infrared spectroscopy (FT-IR) spectra were measured using an Avatar 370 instrument (Nicolet, USA) to indicate the structural changes from the original materials (arginine and glucose) to the resultant CDs. The hydrodynamic sizes and surface charges of the CDs and CDs/pSOX9 nanoparticles were determined using a Zetasizer Nano (Malvern Instruments, Malvern, UK). The fluorescence spectra of the CDs were obtained using a spectrofluorophotometer (RF-5301PC, SHIMADZU), and the absorbance spectra were recorded using a UV-Vis spectrophotometer (UV-2550, SHIMADZU, Japan).

### Agarose gel electrophoresis

The binding of pDNA with CDs was evaluated using agarose gel electrophoresis. The CDs/pSOX9 nanoparticles were freshly prepared at different weight ratios as described in the section of Preparation of CDs/pSOX9 nanoparticles. After 5 min of incubation at room temperature, 10 µL of the complex solution was mixed with 2 µL of loading buffer. The resultant mixture was loaded onto a 1% agarose gel containing ethidium bromide (0.5 µg/mL) buffer at 80 V for 40 min, and the gel was visualized under a UV transilluminator at a wavelength of 365 nm. The pDNA in this study referred to the pSOX9 unless otherwise specified.

### Cytotoxicity

The *in vitro* cytotoxicity of the CDs/pSOX9 nanoparticles against the MEFs was evaluated using the MTT assay. In brief, the MEFs were seeded into a 96-well culture plate at an initial density of 5 × 10^4^ cells/well and incubated for 24 h under the same conditions used for cell culture. The CDs/pSOX9 nanoparticles were then freshly prepared at different weight ratios (1:1, 10:1, 100:1, 500:1, and 1000:1), followed by dilution with the serum-free medium. 100 µL of the nanoparticle-containing, serum-free medium was added to each well (0.2 µg of pDNA for each well) and incubated for 4 h. After removal of the nanoparticle-containing medium, the cells were incubated with the 10% FBS-containing medium for 72 h. After that, 20 µL of the MTT solution (5 mg/mL) was added to each well and incubated for another 4 h. The medium was then removed, and 100 µL of dimethyl sulfoxide was added to form a formazan crystal salt solution. The absorbance of each well was measured using a microplate reader at 570 nm (Epoch, BioTek, USA). The PEI modified CDs (PEI-CDs)/pSOX9 complexes were prepared as previously described^19^ and used as a control in the cytotoxicity assay as well as the subsequent transfection evaluation.

### Localization of complexes

The intracellular distribution of the complexes was evaluated using the confocal microscopy. The MEFs were seeded into 24-well culture plates at an initial density of 2.5 × 10^5^ cells/well and incubated for 24 h under the same conditions used for cell culture. The nucleic acid fluorescent dye YOYO-1 was used to label free plasmid DNA and the CDs/pSOX9 nanoparticles. The YOYO-1-labeled nanoparticles and plasmid DNA were added to the respective wells, and the plates were slightly shaken to create a uniform mixture. After incubation at 37 °C in 5% CO_2_ for different durations (4, 8 and 24 h), the cells were fixed with 4% paraformaldehyde and then visualized using a confocal laser microscope (Leica, DMI6000B, Germany) with three solid-state lasers (405, 488, and 514 nm).

### Pathways for cellular uptake of the CDs/pDNA complexes

To obtain a preliminary understanding of the mechanisms of the CDs internalization, four inhibitors were used to examine the cellular uptake pathways of the CDs/pSOX9 nanoparticles, including filipin III, glucose, 5-(*N*,*N*-dimethyl)-amiloride (DMA), and chlorpromazine hydrochloride (CPZ). The inhibitory functions and concentrations of these inhibitors are summarized in Table 1. Following incubation with these inhibitors at 37 °C for 2 h, the culture medium in each well was replaced with 10% FBS-containing DMEM/F12 medium. After an additional 24 h, the cells were observed using a fluorescence microscope (Leica, DMI6000B, Germany).

**Table 1:** Inhibitors and their Function and Concentration.

Name	Function	Final concentration
Glucose	Inhibits clathrin-dependent endocytosis	0.45 M
CPZ	Inhibits clathrin-dependent endocytosis	10.0 μg/ml
DMA	Inhibits the Na^+^/H^+^ exchange required for macropinocytosis	10 μM
Filipin III	Blocks caveolae/raft-mediated endocytosis	1 μg/ml

The intracellular distribution of the complexes was evaluated using the flow cytometry (FCM). The MEFs were seeded into 6-well culture plates at an initial density of 2.5 × 10^5^ cells/well and incubated for 24 h under the same conditions using cell culture. The YOYO-1-labeled nanoparticles and plasmid DNA were added to the respective wells, and the plates were slightly shaken to create a uniform mixture. After incubation at 37 °C in 5% CO_2_ for 24 h), the cells were viewed under a FCM (BD 6 plus) with three solid-state lasers (488 nm).

### *In vitro* transfection

The MEFs were used to evaluate the transfection efficiency of the CDs/pSOX9 nanoparticles. Specifically, the MEFs were seeded into 96-well culture plates at an initial density of 5 × 10^4^ cells/well and incubated for 24 h under the same conditions used for cell culture. Four hours prior to transfection, the medium in each well was replaced with 100 µL of serum-free medium. The CDs/pSOX9 nanoparticles (0.2 µg of pDNA for each well) at various weight ratios (1:1, 50:1, and 100:1) were added to the wells. The transfection reagents PEI (25 kDa) and Lipofectamine2000 were used as positive controls according to the procedures provided by the manufacturers. After incubation at 37 °C in 5% CO_2_ for 4 h, the medium in each well was replaced with 100 µL of 10% FBS-containing medium, and the cells were incubated for another 72 h. After that, the medium was collected and centrifuged for 5 min at 1500 rpm to obtain the supernatant. The expression level of SOX9 was quantified using the ELISA kit according to the manufacturer’s instructions, and the plate was read at 450 nm using a microplate reader.

### MEFs differentiation into cartilage cells

The MEFs were seeded on a 24-well plate at an initial density of 2.5 × 10^5^ cells/well and incubated for 24 h to obtain a confluence of 60- 70%. The cells were then transfected with the CDs/pSOX9 nanoparticles at a ratio of 40:1 (0.8 µg of pSOX9 per well) as presented in section *In vitro* transfection. The medium was replaced every two days. The cells were transfected once when the samples were collected on day 3, twice when collected on day 7 and four times when collected on day 14 and were fixed with 4% paraformaldehyde at 4 °C for subsequent evaluation. The samples fixed on day 14 were visualized using a confocal laser microscope with three solid-state lasers (405, 488 and 514 nm).

Immunolocalization of collagen II expression in the transfected MEFs was performed using a commercial streptavidin-biotin complex (SABC) kit (Boster, Wuhan, China). According to the manufacturer’s instructions, each sample was incubated in normal goat serum for 20 min at room temperature, followed by incubation with rabbit polyclonal antibodies against mouse collagen II (Abcam, Cambridge, MA) at a dilution of 1:500 in PBS overnight at 4 °C. After washing three times with PBS, the cells were incubated with biotinylated goat anti-rabbit IgG for 20 min at 37 °C. Then, the cells were exposed to SABC and stained with 3, 3-diaminobenzidine (DAB) (Boster, Wuhan, China). For the control staining, PBS was used instead of the primary antibody.

The metachromatic dye toluidine blue was used to stain cartilage cells generated from the transfected MEFs. Toluidine blue (Toluidine Blue O, Sigma-Aldrich; 1% NaCl, pH = 2.3) was added to the fixed plates for 5 min at ambient temperature^40^. Western blotting of collagen II was tested in transfected MEFs on days 3, 7 and 14, while un-transfected MEFs was used as a control.

### Immunogenicity test

Twenty (20) male mice were given 0.2 ml CDs/pDNA by intravenous injection and observed under 24 h. The concentration of CDs was 100 fold of cell transfection. After 24 h, the mice eyeballs were extracted and whole blood was taken and the blood cells counted.

### Statistical analysis

All data were presented as mean ± standard deviation (SD). The Students t-test was performed to determine the significance (a p < 0.05 was considered statistically significant) of the differences between the selected groups using the statistical software of SPSS 14.0 (SPSS company, USA).

## Results and Discussion

### Characterization of CDs

**Figure 1:**
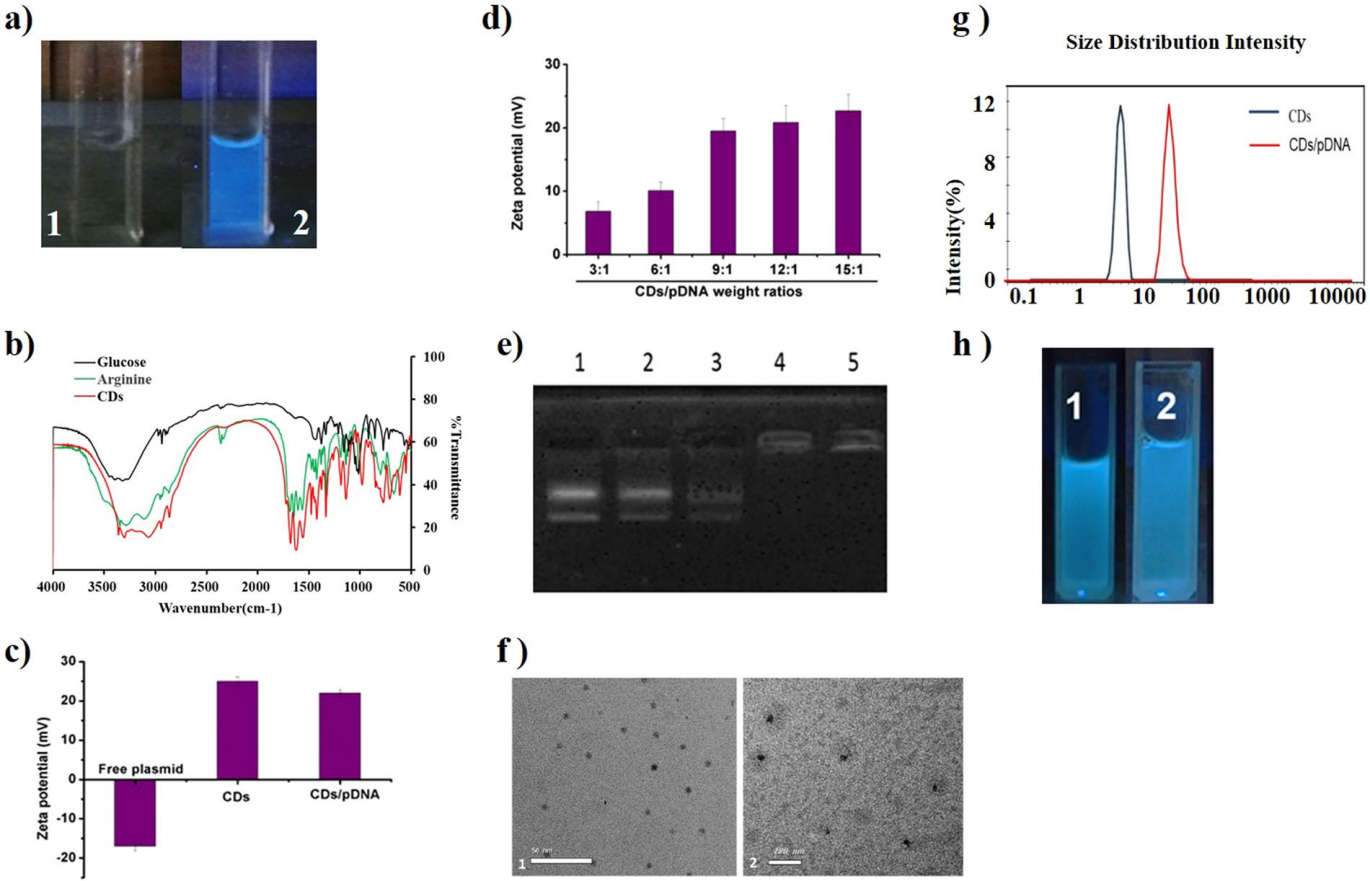
Characterization of CDs and CDs/pDNA complexes. (**a**) Aqueous CDs solutions under UV light at 365 nm: 1, double distilled water; 2, CDs solution; (**b**) FT-IR spectra of glucose, arginine and CDs; (**c**) Zeta potential of free plasmid, CDs and CDs/pDNA complexes; (**d**) Zeta potential of CDs/pDNA complexes prepared at CDs/pDNA weight ratios of 3:1, 6:1, 9:1 12:1, and 15:1; (**e**) Agarose gel electrophoresis: CDs/pDNA complexes at different weight ratios (1:1, 3:1, 6:1, 9:1, and 12:1, from left to right); (**f**) TEM images: 1, CDs; 2, CDs/pDNA complexes; (**g**) Particle size distribution of CDs and CDs/pDNA complexes (2:1); (**h**) Aqueous CDs before (1) and after (2) complexation with pDNA under UV light at 365 nm. Values are the means ± SD of three repeated experiments.

The CDs exhibited excellent water-soluble properties^41^ with blue luminescence emission (Fig. 1a). The FT-IR spectra analysis demonstrated that the CDs combined the characteristic IR absorptions from the both starting materials (arginine and glucose): the broad absorption band ranging from 3000 cm^-1^ to 3410 cm^−1^ (the vibration of N-H and O-H groups), 1459 cm^−1^ (bending vibration of –CH_2_–),1377 cm^−1^ (symmetrical deformation vibration of –CH_3_) in both arginine and glucose (Fig. 1b). Meanwhile, a new, strong peak at 1630 cm^−1^ which may be attributed to the C=O groups emerged in the CDs’ spectrum (Fig. 1b), indicating the chemical reactions occurred during the preparation of the CDs and the arginine residues have been successfully grafted onto the surface of the CDs *in situ*. Previous reports have shown that surface passivation plays a key role in the combination of CDs and pDNA^23^. PEI which has usually been used as a gene carrier in spite of a certain level of cytotoxicity has also been widely employed as a passivating agent during the preparation of CDs^20,42^. The result of zeta potential test revealed that the CDs produced in this study possessed a positive charge (25.4 ± 0.3 mV) (Fig. 1c) without surface passivation. This is likely due to the presence of amino groups on the CDs surface.

The positive charge of the CDs is required for the combination with the negatively charged DNA. To test this, Zeta potential and agarose gel electrophoresis was performed to examine the effective combination of CDs and pDNA with a series of weight ratios. The finding demonstrated that the distance of pDNA migration across the agarose gel gradually decreased with an increasing CDs/pDNA weight ratio (1:1, 3:1, 6:1, 9:1 and 12:1); when this ratio increased to 9:1, the pDNA was completely retained in the original well, suggesting minimum effective weight ratio that prevented DNA migration was 9:1 (Fig. 1e). The CDs/pDNA complex still cannot keep pDNA in the original location until the 9:1 weight ratio, and this could be due that one pDNA molecule might combined with several CDs while other pDNA are still in free form. So it is observed that all zeta potential of the complex are positive, meaning the amount of CDs is not sufficient to coat all the pDNA until the 9:1mass ratio is attained. Moreover, the CDs/pDNA nanoparticles (9:1) also showed a positive charge (21.8 ± 0.2 mV) (Fig. 1d), which would facilitate the cellular uptake of the CDs/pDNA nanoparticles.

The quantum yield was determined by employing quinine sulfate (quantum yield of 54%) as a standard sample, and the result showed that the CDs enjoyed a quantum yield of 12.7%, which was comparable to previous reports^43^.

The morphology of the CDs before and after combination with the pDNA was observed with TEM. As shown in Fig. 1f, the CDs (panel 1) and CDs/pSOX9 nanoparticles (panel 2) were uniformly distributed and had a spherical or elliptical morphology. The particle size determination by dynamic light scattering technique showed that these CDs (with diameters within the range of 1–7 nm, Fig. 1g) and CDs/pSOX9 nanoparticles (10–30 nm in diameter, Fig. 1g) both possessed an ultra small particle size, which is consistent with the TEM analysis. When positively charge CDs solution was mixed with negatively charge pDNA solution a homogeneous mixture is formed. The two oppositely charged ions combined to form nanoparticles. The addition of the two solutions resulted in charge neutralization, bridging and aggregation and this might have led to size decrease. Hence, the particle size ratio reduced from 3:1 to 9:1 (CDs:pDNA) Thereafter, with more addition of CDs, the levels of positively charged CDs increase with respect to negatively charged pDNA resulting in low charge neutralization and static repulsion. The particle size therefore increased from the ratios 9:1 to 15:1(S2). Additionally, the combination of CDs and pDNA generated a strong fluorescence intensity that was similar to that of CDs alone (Fig. 1h), indicating that the combination with pDNA did not significantly affect the CDs fluorescence.

### Cytotoxicity

The integration of transfection ability and effective bioimaging into a single CD nano vector requires not only suitable optical and delivery properties but also low cytotoxicity. Previously reported CDs which have been usually been passivated by PEI exhibited dose- dependent cytotoxicity^43^. However, the CDs prepared in this study avoid this problem with no surface passivation.

**Figure 2:**
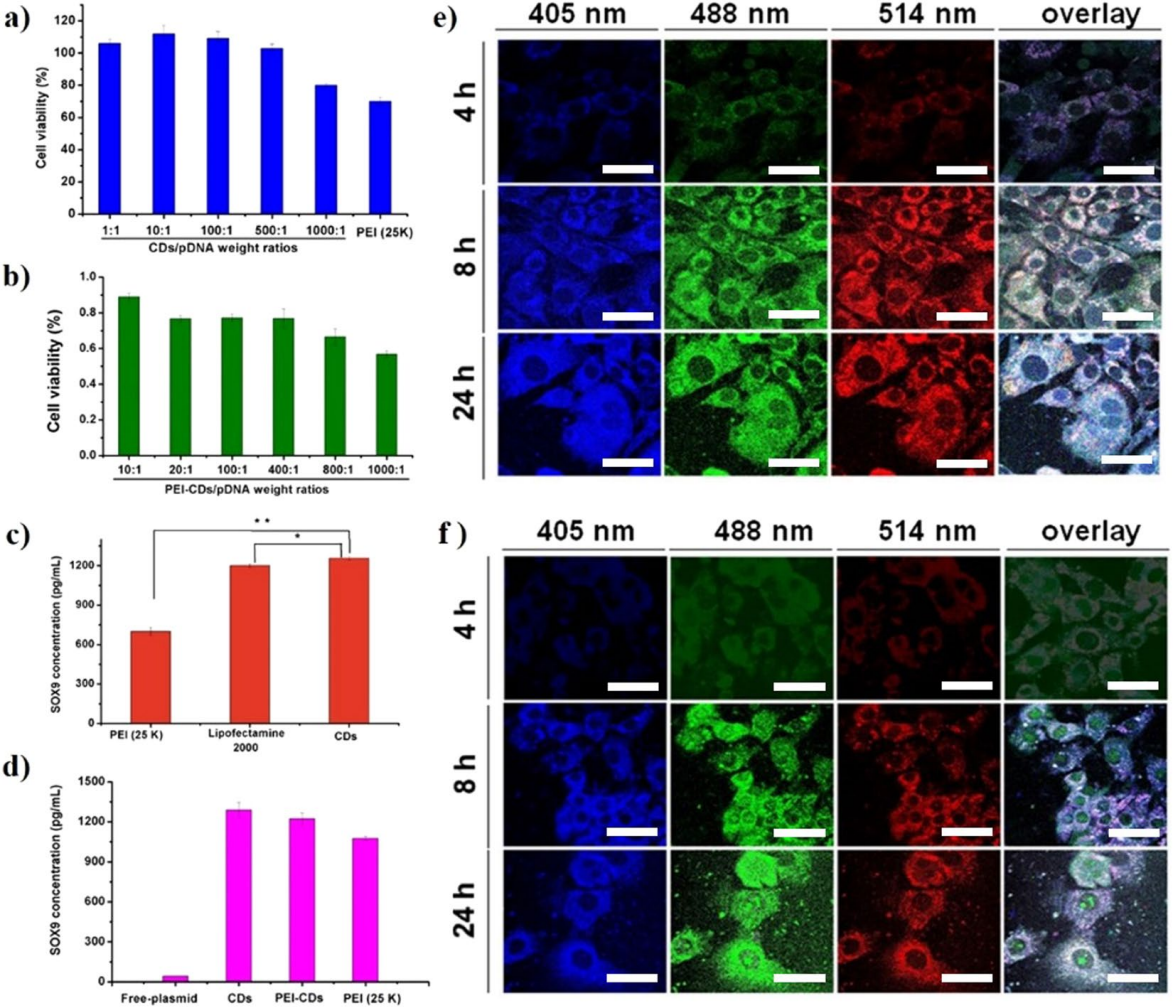
Cytotoxicity assay and transfection efficiency. (**a**) Cytotoxicity of CDs/pDNA nanoparticles formed at various ratios (1:1, 10:1, 100:1, 500:1, and 1000:1) compared with PEI (25 kDa) (**P < 0.01); (**b**) Cytotoxicity of PEI-CDs/pDNA complexes formed at various ratios (10:1, 20:1, 100:1, 400:1, 800:1, and 1000:1); (**c**) *In vitro* gene transfection efficiency of CDs/pDNA nanoparticles compared with PEI (25 kDa)/pDNA and Lipofectamine 2000/pDNA (*P < 0.05; **P < 0.01); (**d**) Comparison of the transfection efficiency among CDs/pDNA nanoparticles, PEI-CDs/pDNA complexes, PEI (25 kDa)/pDNA and free plasmid DNA; (**e**) Real-time imaging of CDs/pDNA complexes following transfection of UCDSCs. Similar conditions were applied in this case; (**f**) Real-time imaging of CDs/pDNA complexes with YOYO-1-labeled DNA following transfection of MEFs. The 405, 488 and 514 nm cell columns were observed 4, 8, and 24 h after transfection. Scare bars = 5 μm. Values are the means ± SD of three repeated experiments.

To evaluate the cytotoxicity of the CDs, the relative viabilities of MEFs exposed to CDs were determined using an MTT assay. The PEI-CDs and PEI (25 kDa) were used as controls. As shown in Fig. 2a, the CDs/pSOX9 nanoparticles exhibited no cytotoxicity when the CDs/pDNA weight ratio was increased from 1:1 to 500:1 (far surpassed the weight ratio used in transfection assay), followed by an acceptable dose-dependent cytotoxicity. Moreover, apoptosis was not observed for the CDs/pSOX9 nanoparticles at weight ratios of 1:1, 10:1, 100:1 and 500:1 (Fig. 2a). In contrast, both the PEI-CDs and PEI (25 kDa) showed significantly decreased cell viability when compared with the CDs (Fig. 2b). The cell viability of the CDs/pSOX9 nanoparticles was superior to that of PEI (25 kDa) even at a CDs/pSOX9 weight ratio of 1000:1. These results indicated that the CDs prepared in this study had an excellent biocompatibility.

### *In vitro* transfection

With the proper ratio of CDs/pDNA (S2), the CDs/pSOX9 nanoparticles with a weight ratio of 9:1 were used in the subsequent experiments. The PEI (25 kDa) and Lipofectamine2000 were used as controls, and the PEI-CDs were employed in parallel experiments.

The results revealed that the MEFs transfected with the CDs/pSOX9 nanoparticles had a SOX9 expression level comparable to that of the Lipofectamine2000, which was significantly higher than that of the transfection reagent PEI (25 kDa) (Fig. 2c). Furthermore, the CDs/pSOX9 nanoparticles possessed considerably enhanced transfection efficiency than the PEI-CDs/pDNA complexes (Fig. 2d). PEI, a widely used cationic polymer gene carrier easily attached into cells, has a high transfection together with high cytotoxicity. One way due that PEI make cell membrane broken which lead to cell necrosis(immediate); another the mitochondrial membrane breakage of the cell by swallowing PEI, the cell eventually lead to apoptosis (delay). Compare with PEI, CDs with cationic and small size enhances pDNA attached to cell membrane without any cytotoxicity. These results suggested that the CDs prepared in this study could serve as a novel, highly efficient non-viral vector for gene delivery.

### Subcellular localization

The dual-functional CDs prepared in this study not only possessed high transfection efficiency but also had the self-imaging ability. Considering that the CDs might not be able to enter nuclei, the green fluorescent nucleic acid dye YOYO-1 was employed to track and locate the exogenous pDNA within the cells. With the laser confocal fluorescence microscopy, the translocation process of the CDs/pSOX9 nanoparticles and the YOYO-1-labeled CDs/pSOX9 nanoparticles in MEFs was observed 4, 8 or 24 h after transfection. As shown in Fig. 2e,f, the plasmid DNA was clearly observed in the cytoplasm 8 h after transfection with the CDs/pSOX9 nanoparticles when exicted at the three wavelengthes (405, 488, and 514 nm), but there was no clear indication of nucleic localization of the exogenous plasmid SOX9 (Fig. 2e). On the other hand, the YOYO-1-labeled CDs/pSOX9 nanoparticles-transfected group showed obvious nucleic localization of the exogenous plasmid SOX9 as indicated by the green fluorescence emitted by YOYO-1 (Fig. 2f). This result indicated that CDs could not enter nuclei, but the exogenous plasmid DNA could be successfully released from the CDs/pDNA nanoparticles and enter nuclei where the exogenous gene could be effectively expressed.

### Cellular uptake

**Figure 3:**
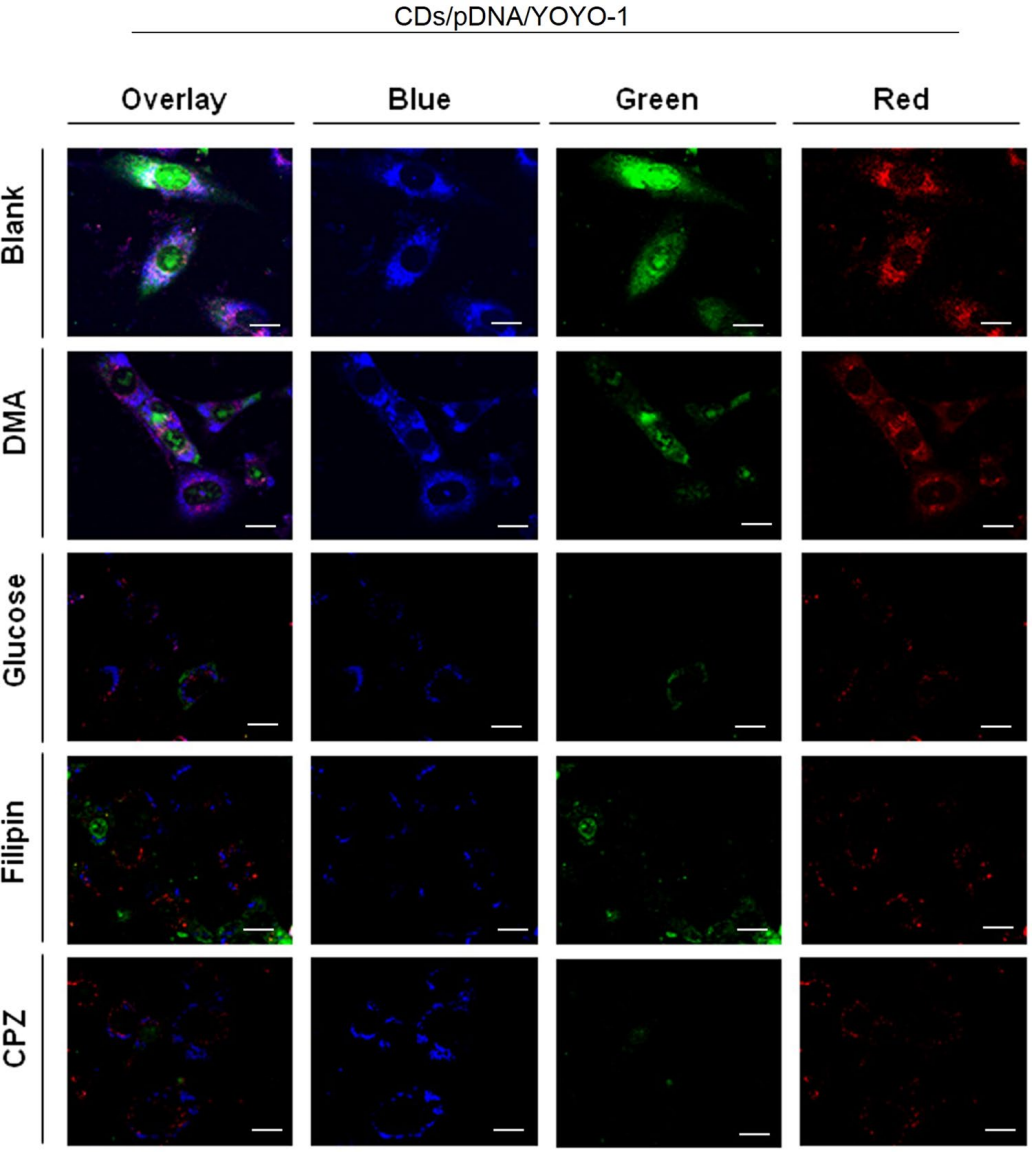
Cellular uptake analyses. MEFs observed using laser scanning confocal microscopy at excitation wavelengths at 405, 488 and 514 nm for blue, green and red fluorescence, respectively, following inhibition with filipin III, glucose, 5-(*N*,*N*-dimethyl)-amiloride (DMA), and chlorpromazine hydrochloride (CPZ). Scare bars = 20 μm.

**Figure 4:**
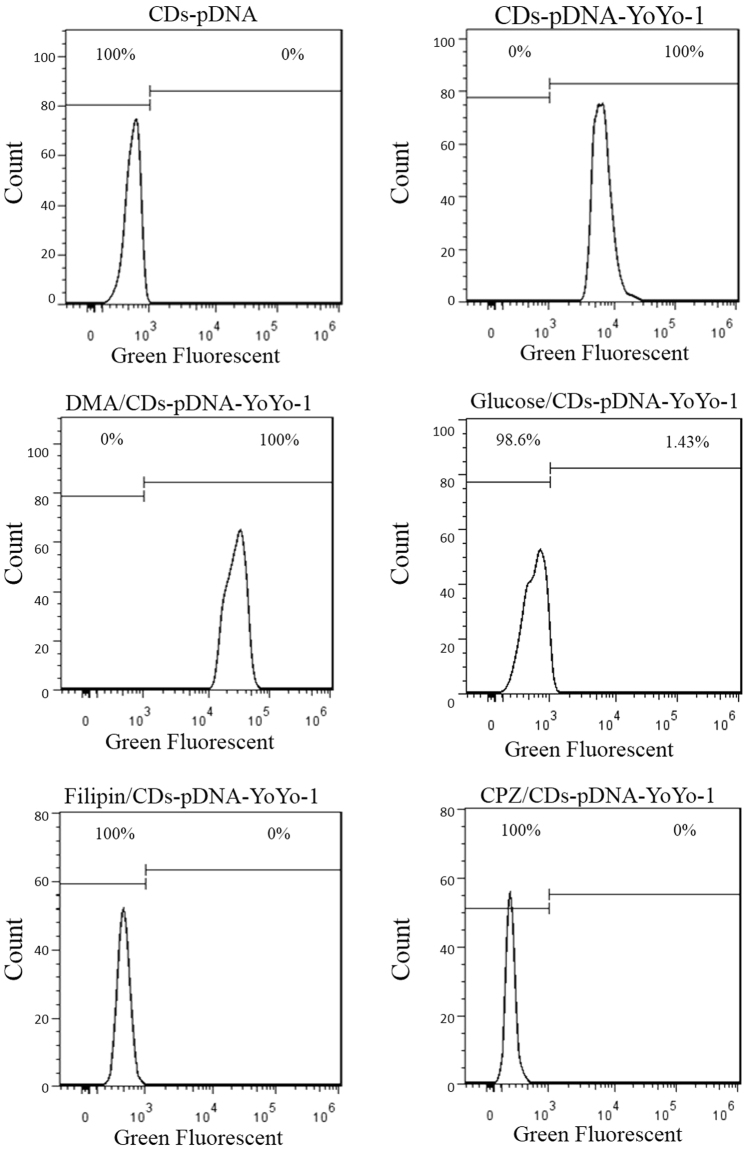
Flow cytometry analyses. MEFs observed using Flow cytometry at excitation wavelengths 488 for green fluorescence, respectively, following inhibition with filipin III, glucose, 5-(*N*,*N*-dimethyl)-amiloride (DMA), and chlorpromazine hydrochloride (CPZ).

The mechanisms of the internalization of the CDs/pDNA complexes were investigated by employing four cellular uptake inhibitors: filipin III, glucose, 5-(*N*, *N*-dimethyl)-amiloride (DMA), and chlorpromazine hydrochloride (CPZ). It is well recognized that filipin III inhibits caveolae-mediated endocytosis^44^, glucose and CPZ are typical inhibitors for clathrin-mediated endocytosis^45^, and DMA is known to disrupt macropinocytosis^46^. As shown in Fig. 3, when compared with the blank control, the fluorescence intensity in the cells treated with filipin III, glucose and CPZ was notably decreased, whereas the cells incubated with DMA did not exhibit differences in the fluorescence intensity from the blank control group. As shown in Fig. 4, when cells were cultivated with glucose, filipin III and CPZ, no fluorescence was emitted by the cells while cells treated with DMA exhibited strong fluorescent intensity. These results indicated that both caveolae- and clathrin-mediated endocytosis represent the major cellular uptake mechanisms of the CDs/pDNA complexes, whereas macropinocytosis plays a minimal role. It is likely that multiple endocytosis pathways may lead to the high transfection efficiency of the CDs/pDNA complexes.

### Chondrogenic differentiation

**Figure 5:**
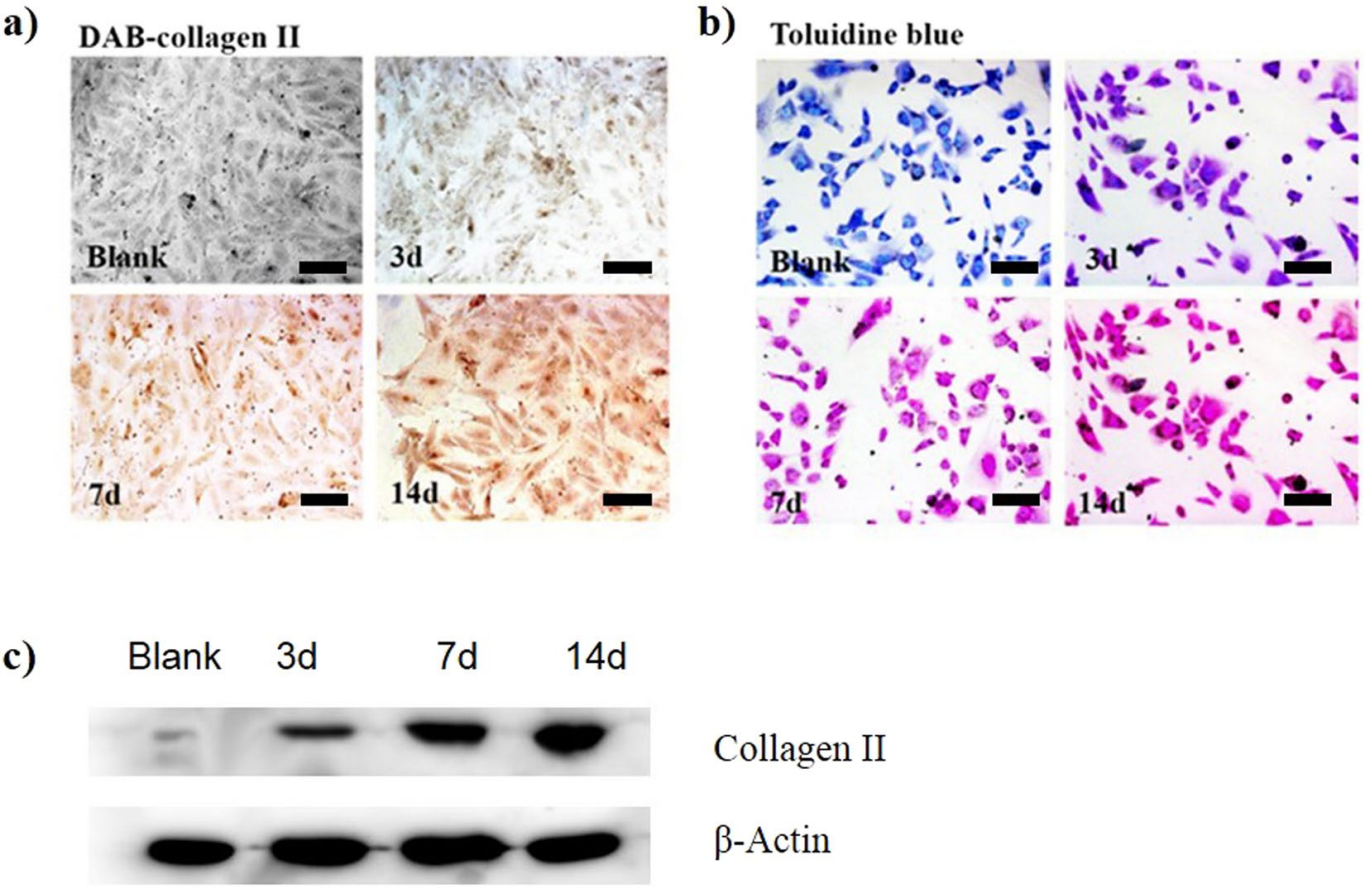
Identification of MEF differentiation into chondrocytes. (**a**) Immunohistochemical staining for collagen II using DAB. Blank: MEFs, 3days-14days; MEFs transfected with CDs/pDNA complexes for 3, 7 or14 days; scare bars = 50 μm. (**b**) Toluidine blue staining. Blank: MEFs, 3 days-14days; MEFs transfected with CDs/pDNA complexes for 3, 7 or14 days; (**c**) Western bolt results of collagen II and GAG, and β-actin was used as control. scare bars = 50 μm.

With the highly efficient expression of SOX9 which is known for induction of chondrogenesis, the chondrogenic differentiation of the transfected MEFs were assessed at various time points (day 3, 7 and 14) during the 2-week culture process. Using DAB staining (Fig. 5a), the CDs/pSOX9 nanoparticles-transfected MEF was positive (shown as increasing color intensity when the incubation time was prolonged) for collagen II marker, whereas untransfected MEFs showed no expression of collagen II (blank). The toluidine blue staining further confirmed the gradual process of cartilage formation as shown by the gradual color change from purple to fuchsia from day 3 to day 14 (Fig. 5b). The similar results of Western blotting (Fig. 5c) can be seen with DAB staining for collagen II. These data demonstrated that the CDs/pSOX9 nanoparticles-transfected MEFs could successfully differentiate into cartilage cells.

**Figure 6:**
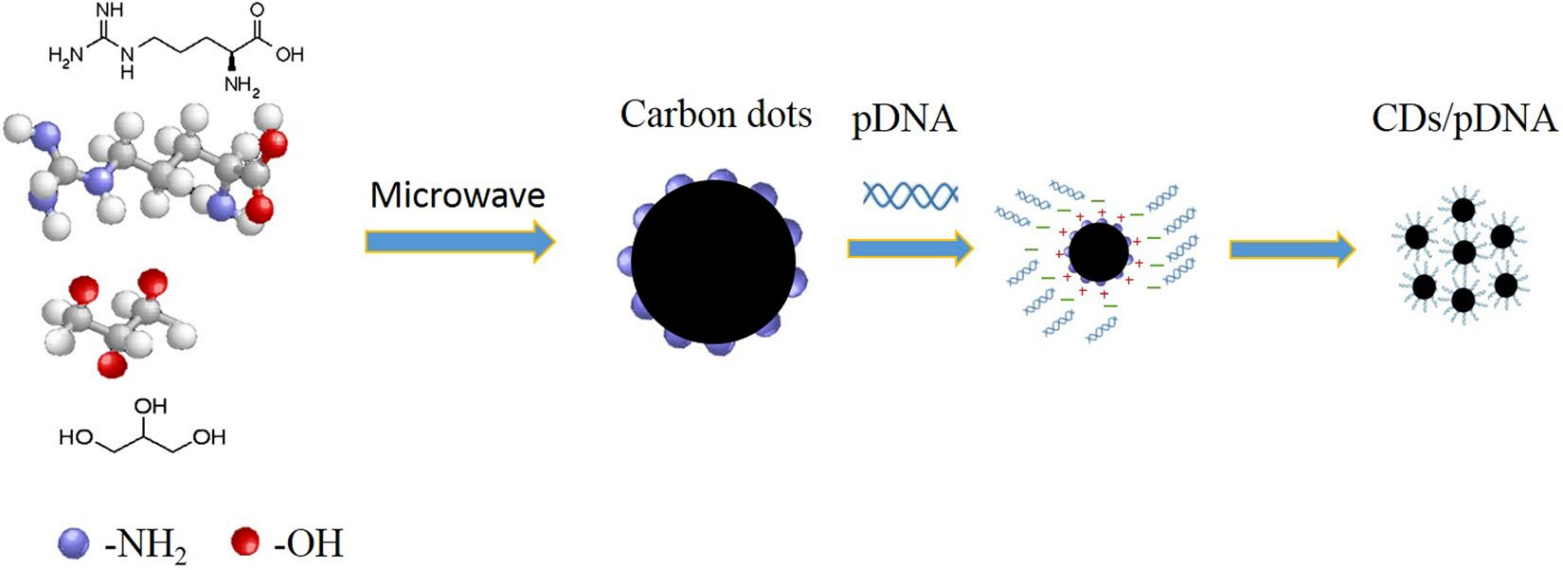
Sketch of the formation of CDs and CDs/plasmid.

### Immunogenicity test

Due to high viral vector immunogenicity and viral recombination, more researchers have been favoring non-viral carriers as they possess properties like noninfectious, unlimited capacity, controlled chemical structure and large preparation. Nevertheless, most of the non-viral carriers are less efficient, and are quickly eliminated from the body after their delivery. We developed a kind of small size (10 nm) gene vector and efficient tracer, which is small enough to escape elimination from the body and possess self-tracking ability. We found that the mice did not show any abnormal behavior, irritant reactions, and unaffected activities like eating. Meanwhile, no blood coagulation or erythrocyte aggregation effect was seen on the mice. The Mice blood cell counts after intravenous treatments with CDs/pSOX9 nanoparticles at concentration of 100 mg/kg for 7days, one dose per day (n = 3). The cells in the CBC (white blood cells, red blood cells, and platelets) have unique functions, which can also be done as a part of an evaluation based on symptoms. The white blood cells are an essential part of the immune system and help the body fight infections. Each different component of the white blood cell (the WBC differential) plays a specific role in the immune system. Red blood cells are essential in transporting oxygen to all the cells in the body to serve their functions. Platelets are a part of the blood clotting system in the body and help in preventing bleeding. The cell counts of CDs/pDNA groups had no appreciable different with normal group. The results showed the biosecurity of CDs (Table 2).

**Table 2:** Mice blood cell counts after intravenous treatment with CDs/DNA for 7days, ones dose per day (n = 10).

Groups	WBC(×10^9^/L)	RBC(×10^12^/L)	Platelets(×10^13^/L)
Saline	5.12 ± 0.98	7.29 ± 1.12	1.28 ± 0.79
CDs/DNA	5.51 ± 1.11	7.13 ± 0.89	1.18 ± 0.52

## Conclusions

In this study, we prepared the CDs with low cytotoxicity by a one-step microwave- assisted pyrolysis of arginine and glucose, which were utilized as a novel, safe, highly efficient, dual-functional (gene delivery and self-tracking) cationic nano gene vector. The synthesized CDs not only had high gene transfection efficiency but also enabled the intracellular tracking of the delivered molecules through tunable fluorescence studies. Furthermore, the CDs/pDNA nanoparticles could be internalized *via* both caveolae- and clathrin-mediated endocytosis and enter nuclei to achieve an effective gene expression. More importantly, the CDs-mediated delivery of plasmid SOX9 could successfully induce the chondrogenesis from the transfected MEFs. Taken together, the judicious application of the dual-functional CDs as both the non-viral gene vector and self-imaging reagent described herein holds a great promise for the non-viral gene delivery, tissue engineering as well as bioimaging.

## Electronic supplementary material

Supplementary Information
