# Supplementary material for: Photoluminescent Cationic Carbon Dots as efficient Non-Viral Delivery of Plasmid SOX9 and Chondrogenesis of Fibroblasts
Source: Sci Rep. 2018 May 4;8:7057. doi: 10.1038/s41598-018-25330-x (PMC5935676; doi:10.1038/s41598-018-25330-x)
Supplement: Supplementary file 1 — Supplementary Information [file 41598_2018_25330_MOESM1_ESM.docx]

**Supplementary**

**Photoluminescent Cationic Carbon Dots as efficient Non-Viral Delivery of Plasmid SOX9 and Chondrogenesis of Fibroblasts**

Xia Cao,1,§ Jianping Wang,1,§ Wenwen Deng,1,§ Jingjing Chen,1,§Yan Wang,1 Jie Zhou,1 Pan Du, 1 Wenqian Xu,1 Qiang Wang,1 Qilong Wang, 1 Qingtong Yu,1 Myron Spector,2 Jiangnan Yu,1 Ximing Xu1, *

1. Department of Pharmaceutics, School of Pharmacy, and Center for Drug/Gene Delivery and Tissue Engineering, Jiangsu University, Zhenjiang 212001, P.R. China
2. Department of Orthopedic Surgery, Harvard Medical School, Brigham and Women’s Hospital, 75 Francis St, Boston, MA 02115, USA

§These authors contributed equally to this work.

* Corresponding Author: Prof. Ximing Xu: Email: [xmxu@ujs.edu.cn](mailto:xmxu@ujs.edu.cn), Tel/Fax: +86-511- 85038451.

**Method**

**In vitro transfection.** The MEFs were seeded into 96-well culture plates at an initial density of 5×10^4^ cells/well and incubated for 24 h under the same conditions used for cell culture. Four hours prior to transfection, the medium in each well was replaced with 100 µL of serum-free medium. The CDs/pSOX9 nanoparticles (0.2 µg of pDNA for each well) at various weight ratios (3:1, 6:1, 9:1, 12:1, 15:1) were added to the wells. After incubation at 37 °C in 5% CO_2_ for 4 h, the medium in each well was replaced with 100 µL of 10% FBS-containing medium, and the cells were incubated for another 72 h. After that, the medium was collected and centrifuged for 5 min at 1500 rpm to obtain the supernatant. The expression level of SOX9 was quantified using the ELISA kit according to the manufacturer’s instructions, and the plate was read at 450 nm using a microplate reader.

**Pathways for cellular uptake of the CDs/pDNA complexes.** To obtain a preliminary understanding of the mechanisms of the CDs internalization, four inhibitors were used to examine the cellular uptake pathways of the CDs/pSOX9 nanoparticles with PI, including filipin III, glucose, 5-(N, N-dimethyl)-amiloride (DMA), and chlorpromazine hydrochloride (CPZ). The inhibitory functions and concentrations of these inhibitors are summarized in Table 1. Following the incubation with these inhibitors at 37 °C for 2 h, the culture medium in each well was replaced with 10% FBS-containing DMEM/F12 medium. After an additional 24 h, the cells were observed using a ﬂuorescence microscope (Leica, DMI6000B, Germany).

**Results and Discussion**

**In vitro transfection.** To determine the optimal weight ratio for in vitro gene transfection, CDs/pSOX9 nanoparticles at various weight ratios (3:1, 6:1, 9:1, 12:1 and 15:1) were assessed. The CDs/pSOX9 nanoparticles at the weight ratio of 9:1 possessed the highest transfection (**S.1**). Therefore, the CDs/pSOX9 nanoparticles with a weight ratio of 9:1 were used in the subsequent experiments.

**Cellular uptake.** The mechanisms of the internalization of the CDs/pDNA/PI complexes were investigated by employing four cellular uptake inhibitors: filipin III, glucose, 5-(N, N-dimethyl)-amiloride (DMA), and chlorpromazine hydrochloride (CPZ). It is well recognized that filipin III inhibits caveolae-mediated endocytosis 44, while glucose and CPZ are typical inhibitors for clathrin-mediated endocytosis 45, with DMA being known to disrupt macropinocytosis 46. As shown in S.1, when compared with the blank control, the fluorescence intensity in the cells treated with filipin III, glucose and CPZ was notably decreased, whereas the cells incubated with DMA did not exhibit differences in the fluorescence intensity from the blank control group. These results indicated that both caveolae- and clathrin-mediated endocytosis represent the major cellular uptake mechanisms of the CDs/pDNA complexes, whereas macropinocytosis plays a minimal role. It is likely that multiple endocytosis pathways may lead to the high transfection efficiency of the CDs/pDNA complexes (**S.2**).

#
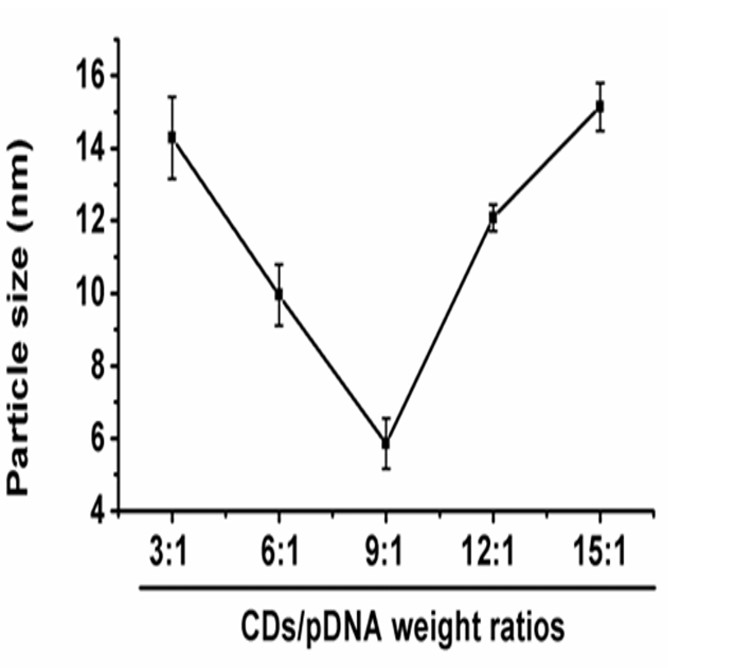


# S. 1 Optimization of the CDs-to-pDNA weight ratio in the CDs/pDNA complexes. (A) Particle size distribution of CDs/pDNA complexes prepared at CDs/pDNA weight ratios of 3:1, 6:1, 9:1 12:1, and 15:1. Values are the means ± SD of three repeated experiments.

#
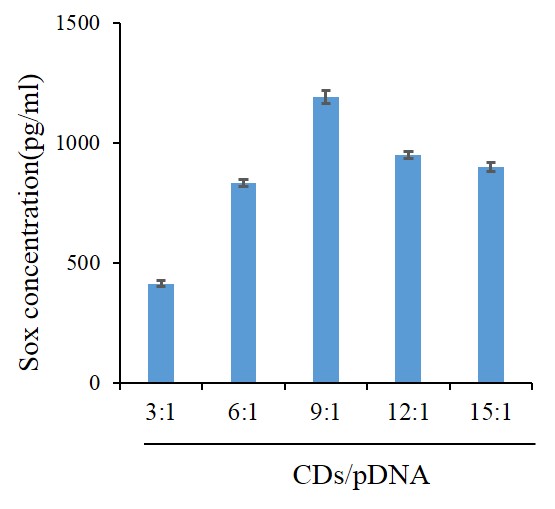


# S. 2 Comparison of the transfection efficiency among different ratio of CDs/pDNA nanoparticles. Values are the means ± SD of three repeated experiments.

#
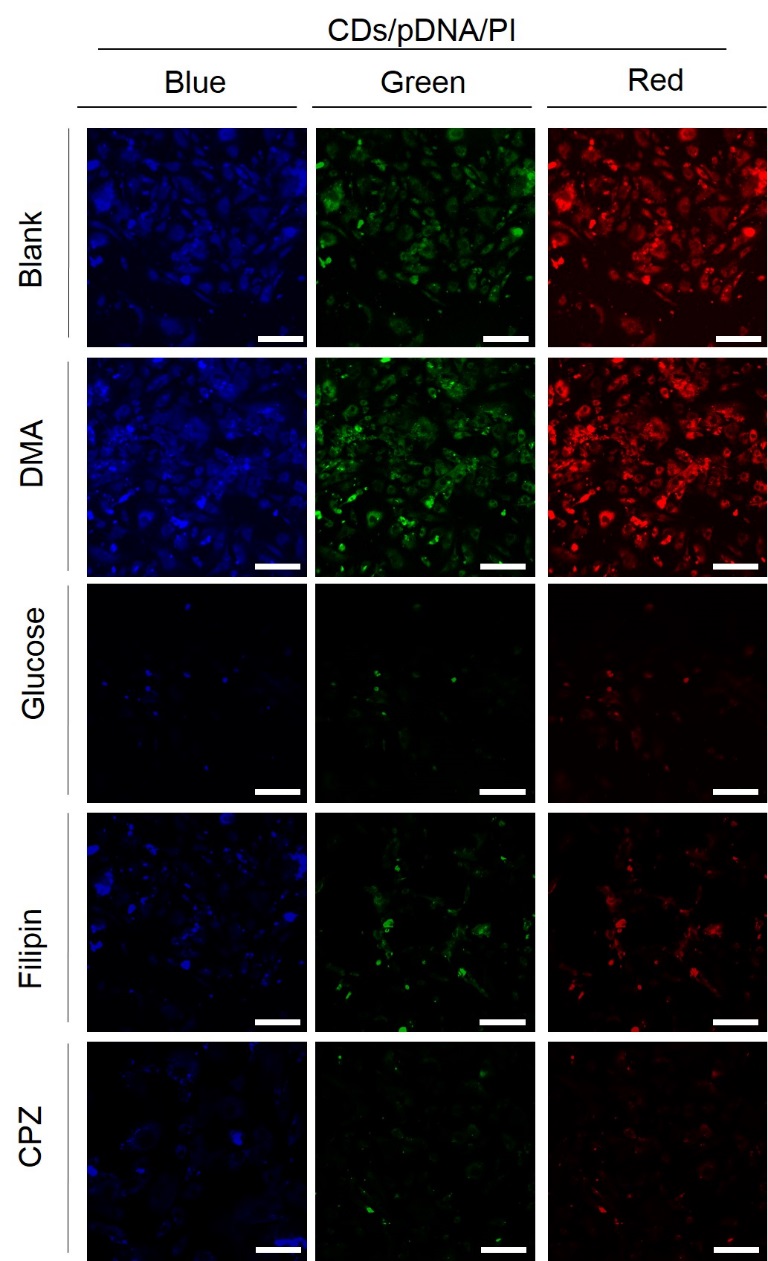


# S. 3 Cellular uptake of CDs/pDNA. MEFs were observed at 405, 488 and 514 nm after transfection under a fluorescent microscopy (Nikon, Ti-E, Japanese). Scare bars=50 μm.

#
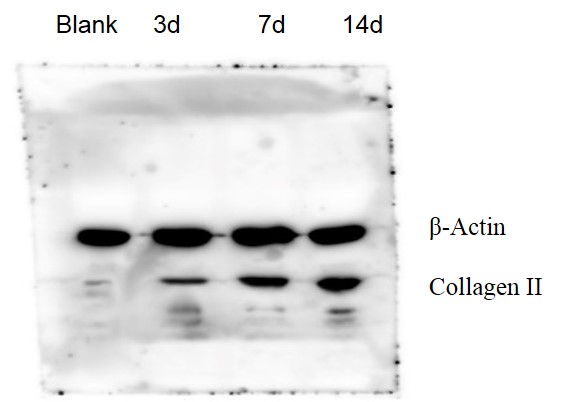


# S.4 Full-length membrane of Western blot
